# *SCN5A*-1795insD founder variant: a unique Dutch experience spanning 7 decades

**DOI:** 10.1007/s12471-023-01799-8

**Published:** 2023-07-20

**Authors:** Virginnio M. Proost, Maarten P. van den Berg, Carol Ann Remme, Arthur A. M. Wilde

**Affiliations:** 1 Department of Clinical Cardiology, Heart Centre, Amsterdam Cardiovascular Sciences, Heart Failure & Arrhythmias, Amsterdam University Medical Centres, location Academic Medical Centre/University of Amsterdam, Amsterdam, The Netherlands; 2 Department of Cardiology, University Medical Centre Groningen, University of Groningen, Groningen, The Netherlands; 3 Department of Experimental Cardiology, Heart Centre, Amsterdam Cardiovascular Sciences, Heart Failure & Arrhythmias, Amsterdam University Medical Centres, location Academic Medical Centre/University of Amsterdam, Amsterdam, The Netherlands

**Keywords:** SCN5A, Overlap syndrome, Sudden cardiac death, Brugada Syndrome, LQT3, Cardiac conduction disease

## Abstract

The *SCN5A*-1795insD founder variant is a unique *SCN5A* gene variant found in a large Dutch pedigree that first came to attention in the late 1950s. To date, this is still one of the largest and best described *SCN5A *founder families worldwide. It was the first time that a single pathogenic variant in *SCN5A* proved to be sufficient to cause a sodium channel overlap syndrome. Affected family members displayed features of Brugada syndrome, cardiac conduction disease and long QT syndrome type 3, thus encompassing features of both loss and gain of sodium channel function. This brief summary takes us past 70 years of clinical experience and over 2 decades of research. It is remarkable to what extent researchers and clinicians have managed to gain understanding of this complex phenotype in a relatively short time. Extensive clinical, genetic, electrophysiological and molecular studies have provided fundamental insights into *SCN5A* and the cardiac sodium channel Nav1.5.

**Supplementary Information:**

The online version of this article (10.1007/s12471-023-01799-8) contains supplementary material, which is available to authorized users.

*“… And so the heart begins to beat with desires at once heroic and tender, we feel that we are on the threshold of the wonders awaiting us further on. As yet we do not see them, that is true—but it is certain, absolutely certain that one day we shall reach them (Dino Buzzati—The Tartar Steppe) …”* [1]

This passage, quoted from the masterpiece of Dino Buzzati (1940), reflects the current mindset in the search for a better understanding of and cure for inherited arrhythmia syndromes. Consistent with the topic of Buzzati’s book, which circulates around the absurdity of human existence and its constant need for routine, thirst for glory and fulfilment, we as doctor-scientists gladly embrace this reality as it will guide us to new discoveries.

This brief review revolves around one of the largest and best described *SCN5A *founder families. This Dutch family demonstrated for the first time that the presence of a single *SCN5A* variant (1795insD, leading to insertion of aspartic acid at position 1795) is sufficient to cause an (*SCN5A*) overlap syndrome of cardiac sodium channel disease [2]. Affected family members displayed clinical signs of Brugada syndrome (BrS), long QT syndrome type 3 (LQT3) and progressive cardiac conduction disease (PCCD), which were previously considered to be separate entities. We will guide the reader past the historic events towards the present day, thereby discussing the genetic, molecular and functional insights and the clinical consequences for affected patients (Fig. 1). By virtue of the very large family pedigree, several other observations have been made, adding to our current understanding of this exceptional founder variant and ion channel disease at large. This literature review is a follow-up to an earlier detailed overview published in the *Netherlands Heart Journal* [3].

**Fig. 1:**
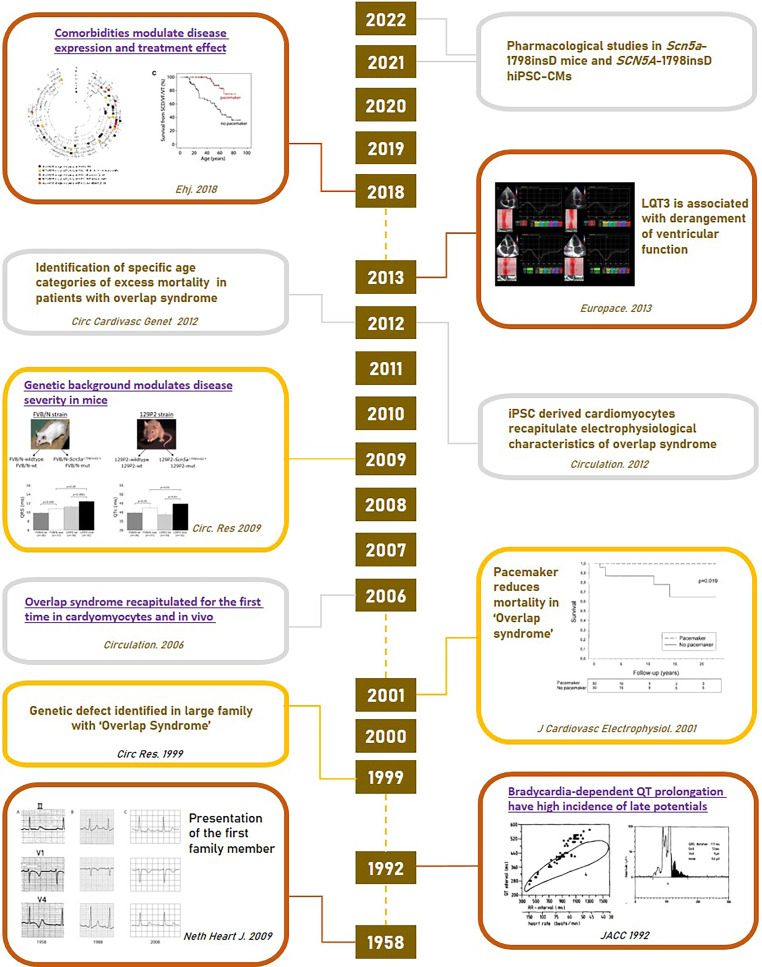
Timeline of major discoveries and achievements in explaining *SCN5A*-1795insD overlap syndrome. These figures were reprinted from (from *top* to *bottom*): Rivaud et al. [7]; Hummel et al. [23]; Van den Berg [6]; Tobé et al. [4]; Postema et al. [3]

## Dutch *SCN5A*-1795insD founder family

In the late 1950s, a large family characterised by premature (mostly nocturnal) sudden cardiac death (SCD) came to the attention of our colleagues in Groningen, the Netherlands [4]. The following series of events were initiated after an abnormal electrocardiogram (ECG) was discovered in a 16-year-old boy during a routine sports examination. The attending physician noticed marked repolarisation abnormalities, which, interestingly, largely disappeared at higher heart rates [4].

The family history was even more intriguing. Several relatives suffered from SCD, urging a comprehensive cardiac evaluation of the remaining family members. This cardiological assessment uncovered a surprising and never seen before cardiac phenotype, with extensive variability in type and severity. Some affected individuals displayed excessive QT interval prolongation at slow heart rates, especially during nighttime as compared with daytime, which was indicative of LQT3 [5]. Others showed right precordial ST-segment elevation, indicative of BrS, and conduction abnormalities at all cardiac levels. While QT prolongation was already present shortly after birth, the ST-segment elevation typically only became apparent after several decades. Of note, atrial fibrillation or dilated cardiomyopathy was typically not observed.

In summary, the affected patients displayed ECG characteristics of BrS, LQT3 and CCD, either in isolation or in combinations thereof [2, 6]. From a historical perspective it’s noteworthy that in the late 1950s, long QT syndrome (LQTS), let alone BrS, was not established as a cardiac disease. Extensive genealogical research and thorough family screening uncovered a very large pedigree, dating back to the 18th century. By 2018, clinical data were available on more than 400 family members [7].

## Overlap explained; how did we get there?

Prior to the identification of the Dutch founder family, BrS and CCD had been shown to be the phenotypes of loss-of-function *SCN5A* mutations, with gain-of-function variants leading to LQT3. Hence, the myriad of clinical arrhythmia syndromes displayed in this family, comprising both BrS/CCD and LQT3, could not be explained by a typical gain-of-function or loss-of-function mechanism, and it was difficult to comprehend that this phenotypic duality was associated with the presence of a single *SCN5A* variant.

It was not until 1999 that the clinical phenomena were attributed to one pathogenic variant in *SCN5A* (1795insD) [2]. Detailed studies of this variant, initially in heterologous expression systems, ‘mapped’ the 2 opposing effects to different phases of the action potential (AP). Whole-cell recordings from *Xenopus* oocytes identified a loss of peak sodium current, predicted to reduce upstroke velocity during phase 0 of the AP (loss-of-function feature). However, these studies did not identify a persistent inward current, nor did they reveal gating defects that could explain the gain-of-function feature [2]. When expressed in HEK 293 cells, *SCN5A*-1795insD channels displayed enhanced slow activation, thereby reducing peak sodium current predominantly at rapid stimulation rates [8]. In the same study, we detected an increased persistent (late) sodium current during sustained depolarisation, typical for the LQT3 phenotype [8]. The observed differences between these cell systems could be explained by the fact that the mammalian HEK 293 cells are more similar to cardiomyocytes in terms of regulatory pathways and interacting proteins; moreover, they allow for improved voltage-clamp conditions and hence more robust electrophysiological characterisation.

As investigations of the mutant channels using these heterologous expression systems could still not fully recapitulate the clinical phenotype, we continued to investigate the mutation in genetically engineered mice carrying the mouse homologue of the human mutation *SCN5A*-1795insD (in mice: *Scn5a*-1798insD^/+^). Indeed, these heterozygous mutant mice recapitulated a large part of the diverse clinical phenotype, including bradycardia, conduction slowing and QT prolongation [9]. Furthermore, epicardial mapping experiments in isolated, Langendorff-perfused hearts showed that conduction was predominantly affected in the right but not left ventricle [9], in accordance with observations in BrS patients [10].

Experiments in *Scn5a*-1798insD^/+^ cardiomyocytes revealed AP prolongation and decreased AP upstroke velocity, consequent to an increase in persistent (late) sodium current and a decrease in peak sodium current, respectively. The enhanced late current occurs during the plateau phase (phase 2) and repolarisation phase (phase 3) of the AP and is more pronounced when these phases last longer (i.e. at lower heart rates). This is in line with the clinically observed longer QT intervals in LQT3 at slow heart rates and normalisation of the QT interval at faster heart rates. Conversely, the reduced peak current density leads to a decreased sodium channel availability predominantly at faster heart rates, in accordance with BrS and CCD. In contrast to the previous transfection studies using HEK 293 cells, sodium current gating properties were largely unchanged in isolated *Scn5a*-1798insD^/+^cardiomyocytes [9].

Overall, these observations confirmed that one single mutation could lead to both gain and loss of sodium channel function, providing the first evidence for an *SCN5A* overlap syndrome. The dual effects of the variant were subsequently also observed in human induced pluripotent stem cell-derived cardiomyocytes (hiPSC-CM) generated from an *SCN5A*-1795insD patient, demonstrating the utility of hiPSC-CMs in modelling even complex ion channel disorders [5].

This experimental work has revealed for the first time sound evidence for the above-described dissociation of the biophysical effects [11]. Today, extensive clinical and biophysical overlaps among the various types of *SCN5A* mutations are known to exist, now referred to as ‘overlap syndromes’ of cardiac sodium channel disease [12–14].

## The clinician’s headache: phenotypic variability

**Fig. 2:**
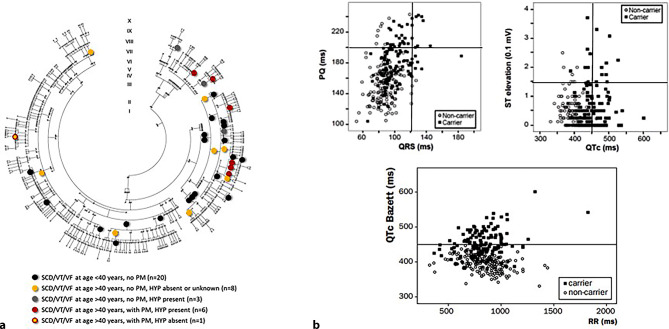
*SCN5A*-1795insD founder population **a** Pedigree, with circles indicating *SCN5A*-1795insD carriers suffering sudden cardiac death (*SCD*), ventricular fibrillation (*VF*) and/or ventricular tachycardia (*VT*). *PM* pacemaker, *HYP* hypertension. **b** Scatterplots showing overlap between mutation carriers (*filled boxes*) and non-carriers (*open circles*) for PQ interval versus QRS duration, ST-segment elevation versus heart rate-corrected QT interval (*QTc*) and QTc versus RR interval. **a** was reprinted from: Rivaud et al. [7]. **b** was reprinted under a CC-BY licence from: Postema et al. [3]

Following the identification of the causal variant, clinical data became available on 164 individuals with the pathogenic variant and 247 relatives not carrying this variant [7], comprising one of the largest founder mutation family pedigrees (Fig. 2a). Naturally, there were phenotypic differences between carriers and non-carriers, most notably in ECG features (Fig. 2b). It can be appreciated that mutation carriers show worst phenotypes and that there is a positive relation between PQ and QRS interval prolongations and ST-segment elevation and heart rate-corrected QT interval (QTc) prolongation. Additionally, bradycardia-dependent QTc prolongation is visible. However, phenotypic variability also existed between carriers. While all carriers showed some sort of ECG abnormality (100% penetrance), some exhibited ECG features of LQT3, others displayed features of BrS or conduction disease and several showed a combination of all 3 phenotypes.

Over the years, it has become evident that the genetic culprit has relatively modest impact on explaining the phenotype variability and the ability to distinguish patients at high risk for cardiac events. The 1795insD phenotype seems best understood as the final common response of the heart to diverse genetic and environmental insults. Known modifiers of disease severity in the inherited cardiac rhythm disorders include age, comorbidities and use of medication, and these most likely also modulated the phenotype in this family. For example, as previously mentioned, age-dependent penetrance of the electrocardiographic characteristics of LQT3, BrS and CCD has been demonstrated in this family [15]. More recently, the potential modulatory role of common comorbidities has become apparent. Indeed, the co-occurrence of hypertension and consequent cardiac hypertrophy was found to increase the age-dependent risk of ventricular arrhythmias and SCD and decrease the efficacy of pacemaker treatment in *SCN5A*-1795insD mutation-positive patients [7]. Conversely, in the absence of these comorbidities, ventricular arrhythmias and even ventricular premature contractions were not seen [6]. This finding was recapitulated in mouse studies: *Scn5a-*1798insD^/+^ mice subject to cardiac overload leading to cardiac hypertrophy displayed more severe conduction disturbances, ventricular arrhythmias and an increased SCD risk compared with wild-type littermates [7].

Besides such factors—and in the absence of compound mutations—the inheritance of other genetic variants, commonly referred to as genetic modifiers, alongside the primary genetic defect, is believed to be a potential cause of inter-individual variability in disease expression. Genetic modifiers may act to exacerbate the severity of the primary genetic defect, leading to clinical presentation in childhood as opposed to adulthood, or may protect a carrier of a primary genetic defect from developing the disease. The first conclusive evidence that intrinsic genetic modifiers influence disease severity in cardiac ion channelopathies was provided by work from our group, when electrophysiological consequences of the *Scn5a-*1798insD^/+^ variant (in mice) were found to be more severe on the 129P2 genetic background compared with FVBN/J mice [16]. By comparing cardiac gene expression between these 2 strains of mice, *Scn4b*, which encodes a sodium channel β‑subunit, was discovered as a potential modifier of conduction disease severity. More recently, in a systems genetics approach on F2 progeny arising from the 129P2 and FVBN/J mouse strains carrying the *Scn5a-*1798insD^/+^ variant, we furthermore identified *Tnni3k* encoding troponin 1‑interacting kinase as a novel modulator of cardiac conduction [17, 18]. These observations strengthen the concept that genetic modifiers are pivotal for disease severity in patients carrying pathogenic variants including the *SCN5A*-1795insD variant.

Genetic variation between individuals takes various forms, with the largest part being explained by single nucleotide polymorphisms (SNPs), which make up about 90% of all human genetic variation. The combination of SNPs identified in large genome-wide association studies (GWASs) conducted in the general population as modulators of heart rate and ECG indices of conduction and repolarisation [19] constitutes an ideal candidate for modulatory effects. Indeed, more recent GWASs established an important role for common genetic variation in susceptibility to LQTS and BrS [20, 21]. Unfortunately, conducting a GWAS in this family is not feasible due to the still relatively small sample size. Interestingly, a polygenetic risk score comprising variants from BrS susceptibility loci correlated with the BrS phenotype in individuals carrying the *SCN5A-*E1784K variant [22]. Thus, it seems feasible that susceptibility loci play a role in the disease variability in this family. These observations have the potential to transform the ‘clinician’s headache’ into a cardiogenetic land of opportunity.

## Arrhythmia mechanisms: 2 separate modes of sudden cardiac death

In a complex phenotype as present in this family, it is a daunting task for physicians to tell who is at risk for serious cardiac events. Thus far, a total of 38 individuals died suddenly or suffered from life-threatening ventricular arrhythmia (Fig. 2a; [7]). The actual mode of death in this family has long been a mystery. Bradycardia-induced polymorphic ventricular tachyarrhythmia, in particular torsades de pointes, was originally suspected, thus pointing to the LQT3 phenotype as the aggressor. This suspicion was supported by occasional observations of (severe) sinus bradycardia and self-terminating torsades de pointes arrhythmias, as well as T‑wave alternans in 3 patients. The observed efficacy of pacemaker treatment [6], aimed at prevention of pauses and the related pause-dependent torsades de pointes arrhythmia, also supported this thought. However, a counter argument is that such arrhythmias were in fact never recorded, despite very extensive Holter recording [6]. It is also possible that bradyarrhythmias, leading to asystole, may be the ultimate event.

Surprisingly, several sudden deaths occurred in patients with a pacemaker after the age of 40 (see Figure S1a in Electronic Supplementary Material), with one 58-year-old female, mutation-positive patient displaying a nocturnal, fast (up to 300 bpm) and apparently polymorphic ventricular tachycardia on pacemaker readout [7]. As discussed in more detail below, it was hypothesised that an age-dependent shift exists, from bradycardia-induced to apparent bradycardia-independent arrhythmias and SCD in older *SCN5A*-1795insD patients, which is possibly mediated by concurrent hypertension and consequent cardiac hypertrophy (see Figure S1b in Electronic Supplementary Material) [7].

**Fig. 3:**
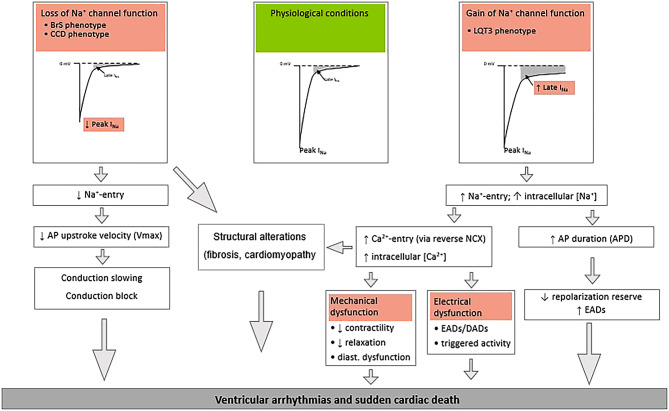
Mechanisms of ventricular arrhythmias and sudden cardiac death in loss and gain of sodium channel function. *BrS* Brugada syndrome, *CCD* cardiac conduction disease, *AP* action potential, *LQT3* long QT syndrome type 3, *NCX* sodium calcium exchanger, *EADs* early after-depolarisations, *DADs* delayed after-depolarisations, *APD* action potential duration

On the cardiomyocyte level, multiple pro-arrhythmic mechanisms of this founder mutation have been observed (Fig. 3). The enhanced late sodium current leads to a persistent influx of sodium ions throughout the entire duration of the AP, depolarising the membrane and prolonging AP duration, thereby increasing the susceptibility to early after-depolarisations, particularly at slow heart rates. This sustained influx of sodium ions may furthermore lead to higher intracellular sodium concentrations within the cardiomyocyte. This may ultimately increase the intracellular calcium concentration secondary to reverse-mode activity of the sodium-calcium exchanger, thereby facilitating the occurrence of delayed after-depolarisations and triggered activity. In cardiomyocytes from *Scn5a-*1798insD^/+^ mice of different strains (see above), a correlation between the magnitude of the late sodium current, extent of sodium and calcium dysregulation and incidence of pro-arrhythmic events was observed [7]. Moreover, pharmacological late sodium current inhibition reversed these pro-arrhythmic features.

Conversely, the decreased peak inward sodium current and consequent lower influx of sodium ions into the cardiomyocyte reduces upstroke velocity of the AP and slows propagation, particularly at high rates. In addition to inducing atrio-ventricular conduction slowing and CCD, this may set the stage for re-entrant ventricular arrhythmias.

Apart from cardiac electrical disease, cardiac structural phenotypes secondary to sodium channel dysfunction have also been reported in *SCN5A*-1795insD patients [23]. Although the exact mechanisms remain to be elucidated, loss of sodium channel function may impact pro-fibrotic pathways and cell adhesion of neighbouring cardiomyocytes, which, in turn, reduces peak sodium current. Thus, loss of sodium channel function not only modulates electrophysiological properties of the heart, but also affects cardiac structure, thereby further predisposing to arrhythmias. Gain of sodium channel function may enhance calcium-dependent structural alterations [24]. Clearly, development of cardiac structural derangements will contribute to arrhythmogenesis in affected patients. Hypertension and subsequent cardiac hypertrophy act synergistically with the biophysical defects caused by the genetic defect in creating a highly arrhythmogenic environment, as we observed in the *SCN5A*-1795insD family [7].

Hypertrophy is associated with alterations in sodium current, dysregulation of intracellular calcium homeostasis and collagen deposition. In support of this finding, similar levels of hypertrophy induced a pro-arrhythmic phenotype in *Scn5a*-1798insD^/+^ mice. Furthermore, increased intracellular calcium concentrations are a well-established pro-arrhythmic feature of hypertrophy and heart failure and may be exacerbated in *SCN5A*-1795insD variant-positive patients in the presence of hypertrophy [7].

Therefore, a reasonable assumption would be that at later age, the arrhythmia mechanism changes from a predominantly bradycardia-dependent repolarisation disorder to a more complex arrhythmogenic substrate, potentially including structural alterations (see Figure S1b in Electronic Supplementary Material).

## Treatment

Up until 1978, there were no treatment options for affected individuals. Although beta-blockers are the cornerstone of LQTS treatment, they were considered to be contraindicated in this family. The hypothesis that bradycardia triggered arrhythmias led to pacemaker implantation as preventive measure for SCD in this family. This strategy, implemented from early infancy onward, had a tremendous impact and initially seemed to be 100% beneficial, as no more deaths occurred in patients treated with antibrady-pacing [6]. In later years, a family tree mortality study—revealing the natural disease course in a time when the disease was not known nor treated—demonstrated that the SCD risk was negligible before the age of 10 years [25]. Based on these data, pacemakers were no longer implanted in early infancy but in early puberty.

While this success was celebrated for the next 23 years, it eventually became apparent that especially the young patients benefited from this therapy. Until 2018, SCD or life-threatening arrhythmias had occurred in 38 family members, 18 of whom were older than 40 years, including 7 patients with a pacemaker [7]. Thus, antibrady-pacing no longer granted complete protection above the age of 40, likely due to the age-dependent change in arrhythmia mechanism described above. Moreover, in several patients who suffered from SCD despite having a pacemaker, ventricular fibrillation or tachycardia was actually documented, and the majority suffered from hypertension and/or left ventricular hypertrophy on autopsy [7]. These observations underlined the necessity for additional treatment strategies in older patients. Notably, the observed effect of comorbidities on disease expression and severity implicated a substantial role for treating hypertension and subsequent hypertrophy. Indeed, observations in *Scn5a*-1798insD^/+^ mice proved that prevention of hypertrophy (by genetic deletion of *Nfatc2*) was sufficient to prevent SCD. Hence, careful monitoring of *SCN5A*-1795insD carriers for hypertension, in addition to aggressive early treatment in order to prevent left ventricular hypertrophy, was henceforth deemed beneficial and part of routine clinical management [7]. In high-risk patients with overt left ventricular hypertrophy and severe electrical disturbances and/or ectopy, implantable cardioverter-defibrillator therapy may be considered.

Inhibition of the chronically enhanced late sodium current, thereby nullifying the detrimental gain-of-function features of the *SCN5A*-1795insD variant—in combination with the above-described therapy—may furthermore be considered. Benefits of this intervention include not only shortening of the AP duration and reduction of repolarisation dispersion, but also prevention of pro-arrhythmic dysregulation of the intracellular sodium/calcium balance. Sodium channel blockers, including lidocaine, mexiletine, flecainide and ranolazine, are not suited for selective late sodium current inhibition [26–28] and may also affect peak sodium current to some extent, thereby potentially worsening conduction abnormalities. Moreover, they can exhibit pro-arrhythmic effects (e.g. flecainide leads to provocation of BrS phenotype), additional off-target effects (inhibition of delayed-rectifier potassium current) or pharmacokinetic properties (short half-life) that further limit clinical utility. Novel sodium channel blockers, eleclazine and GS967, are highly selective for the late sodium current [29–31] and may prove to be more suitable. Indeed, GS967 decreased the late sodium current and AP duration in both *Scn5a*-1798insD^/+^ mouse cardiomyocytes and *SCN5A*-1795insD hiPSC-CMs and had anti-arrhythmic effects without negatively impacting on cardiac conduction [29].

Ideally, pharmacological treatment would be aimed at both restoring the peak sodium current and diminishing the late sodium current. Mexiletine has such abilities: it may acutely inhibit late sodium current, while it has the ability to chronically restore the peak sodium current through its chaperone effect on sodium channel trafficking. Indeed, recent findings indicate potential beneficial effects on the overlap phenotype in *SCN5A*-1795insD hiPSC-CMs [32], prompting further ongoing (clinical) investigations.

## Conclusion and future directions

The extensive clinical, genetic, electrophysiological and molecular insights into the *SCN5A*-1795insD variant gained in the last 20 years have led to a better understanding of and treatment for the Dutch *SCN5A*-1795insD founder family. This review has also accentuated the importance of translational collaborations between different disciplines. Discovering this unique variant and the large founder family allowed us to go into depths we would otherwise never reach. Moreover, the reconstruction of the phenotype in mice and hiPSC-CM allowed us to explore different interventions and ultimately provided more fundamental insights into *SCN5A* and Nav1.5. Furthermore, the observation of an age-dependent shift in arrhythmia mechanism may prove to be crucial for patient management and has emphasised the importance of comorbidities. The focus moved beyond pacemaker therapy, towards the more chronic consequences of this phenotype: stringent treatment of comorbidities and, more notably, educating patients in preventing these preventable diseases. In the future, there may also be a place for further risk stratification, based on genetic background (genetic risk scores) and individualised medical interventions to nullify the effects of the pathogenic variant. Finally, we recommend incorporating diagnostic modalities to map structural heart defects into standard clinical care, in particular echocardiography including tissue imaging and perhaps cardiac magnetic resonance imaging.

Despite our progress, many questions remain unanswered and there are still some fields to (re)discover, both in the present family and patients with *SCN5A* disorders in general. It has become more and more apparent that sodium channel distribution, function and regulation is more complicated than traditionally assumed [24, 33]. *SCN5A* and Nav1.5 are expressed in cell types other than cardiomyocytes, as well as various extracardiac tissues, where their functional role is increasingly recognised; nevertheless, the impact of pathogenic variants in these other cell and tissue types and their impact on arrhythmogenesis (if any) remain to be explored.

In terms of disease variability, the role of comorbidities other than hypertension needs investigation. For example, the influence of metabolic disorders such as diabetes and obesity could modulate disease expressivity in sodium channelopathy, since it is known that these conditions are associated with reduced *SCN5A* expression, decreased peak sodium current and calcium-dependent proarrhythmic events [34].

Finally, non-electrogenic actions of cardiac sodium channels are increasingly recognised, which may have an impact on cardiac structural integrity, thereby also potentially affecting arrhythmogenesis [24]. Abnormal development and cardiac structural abnormalities in homozygous *Scn5a*-1798insD embryos at an early stage before sodium channels become functionally relevant for cardiac electrical activity provided evidence for a non-electrogenic role of Nav1.5 independent of its ion-conducting properties [35]. Such non-electrogenic effects of Nav1.5 dysfunction may affect myocardial structural integrity and cell adhesion, potentially by impacting the many associated proteins that Nav1.5 is known to interact with [33]. Clearly, further elucidation of the complexity of sodium channel (dys)function and *SCN5A *overlap variants will continue to improve clinical care and outcome in affected individuals.

Overall, our research has provided novel (translational) insights, which include the following: (1) elucidation of the underlying disease and arrhythmia mechanisms is essential, ideally through studies in cardiomyocytes and whole heart systems; (2) arrhythmogenesis may be multifactorial, modulated not only by the genetic defect, but also by concomitant structural alterations in the myocardium; (3) the latter may be modulated by environmental factors and comorbidities; (4) ion channel defects may secondarily alter intracellular ionic homeostasis, setting the stage for, for example, calcium-dependent pro-arrhythmia; and (5) these may provide novel targets for therapeutic interventions. These findings are not only applicable to *SCN5A* overlap syndromes but are also potentially relevant for cardiac arrhythmia disorders in general.

## Supplementary Information

**Figure S1 a** Survival curves of *SCN5A*-1795insD mutation carriers and **b** their age-dependent arrhythmia risk, including impact of comorbidities. Fig. 1a is a modified figure from Rivaud MR, Jansen JA, Postema PG, et al. A common co-morbidity modulates disease expression and treatment efficacy in inherited cardiac sodium channelopathy. Eur Heart J. 2018;39:2898–907. Copyright, with permission from Oxford University Press [7]
