# Supplementary figures and images for: SCN5A-1795insD founder variant: a unique Dutch experience spanning 7 decades
Source: Neth Heart J. 2023 Jul 20;31(7-8):263–71. doi: 10.1007/s12471-023-01799-8 (PMC10400486; doi:10.1007/s12471-023-01799-8)

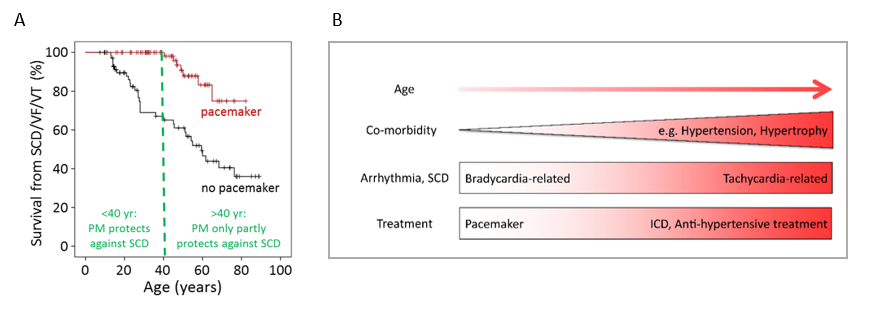

Supplement: Supplementary file 1 — Figure S1 a Survival curves of SCN5A-1795insD mutation carriers and b their age-dependent arrhythmia risk, including impact of comorbidities. Fig. 1a is a modified figure from Rivaud MR, Jansen JA, Postema PG, et al. A common co-morbidity modulates disease expression and treatment efficacy in inherited cardiac sodium channelopathy. Eur Heart J. 2018;39:2898–907. Copyright, with permission from Oxford University Press [7] [file 12471_2023_1799_MOESM1_ESM.docx]
